# Recent Improvement Strategies on Metal-Organic Frameworks as Adsorbent, Catalyst, and Membrane for Wastewater Treatment

**DOI:** 10.3390/molecules26175261

**Published:** 2021-08-30

**Authors:** Arie Wibowo, Maradhana A. Marsudi, Edi Pramono, Jeremiah Belva, Ade W. Y. P. Parmita, Aep Patah, Diana Rakhmawaty Eddy, Akfiny Hasdi Aimon, Aditianto Ramelan

**Affiliations:** 1 Materials Science and Engineering Research Group, Faculty of Mechanical and Aerospace Engineering, Institut Teknologi Bandung, Jl. Ganesha 10, Bandung 40132, West Java, Indonesia; maradhanaa@alumni.itb.ac.id (M.A.M.); jeremiahbelva@gmail.com (J.B.); 2 Research Center for Nanoscience and Nanotechnology, Institut Teknologi Bandung, Jl. Ganesha 10, Bandung 40132, West Java, Indonesia; 3 Department of Chemistry, Faculty of Mathematics and Natural Sciences, Universitas Sebelas Maret, Jl. Ir. Sutami 36, Surakarta 57126, Central Java, Indonesia; edi.pramono.uns@staff.uns.ac.id; 4 Materials and Metallurgy Engineering, Institut Teknologi Kalimantan, Jl. Soekarno Hatta 15, Balikpapan 76127, East Kalimantan, Indonesia; adewahyu27@lecturer.itk.ac.id; 5 Inorganic and Physical Chemistry Research Group, Faculty of Mathematics and Natural Sciences, Institut Teknologi Bandung, Jl. Ganesha 10, Bandung 40132, West Java, Indonesia; aep@chem.itb.ac.id; 6 Department of Chemistry, Faculty of Mathematics and Natural Sciences, Universitas Padjadjaran, Jl. Raya Bandung Sumedang KM.21, Sumedang 45363, West Java, Indonesia; diana.rahmawati@unpad.ac.id; 7 Department of Physics, Faculty of Mathematics and Natural Sciences, Institut Teknologi Bandung, Jl. Ganesha 10, Bandung 40132, West Java, Indonesia; akfiny@fi.itb.ac.id

**Keywords:** adsorption, advanced oxidation processes, membrane, metal-organic frameworks, persistent organic pollutants, photocatalyst, wastewater treatment

## Abstract

The accumulation of pollutants in water is dangerous for the environment and human lives. Some of them are considered as persistent organic pollutants (POPs) that cannot be eliminated from wastewater effluent. Thus, many researchers have devoted their efforts to improving the existing technology or providing an alternative strategy to solve this environmental problem. One of the attractive materials for this purpose are metal-organic frameworks (MOFs) due to their superior high surface area, high porosity, and the tunable features of their structures and function. This review provides an up-to-date and comprehensive description of MOFs and their crucial role as adsorbent, catalyst, and membrane in wastewater treatment. This study also highlighted several strategies to improve their capability to remove pollutants from water effluent.

## 1. Introduction

Clean water is a crucial resource for all living things on Earth. However, massive water contamination and rapid population growth have led to a water scarcity problem. In 2003, Gleick reported that over 1000 million people have limited access to safe drinking water worldwide [1]. Currently, many hazardous contaminants, namely persistent organic pollutants (POPs), are found in industrial discharged water. There are many organic pollutants which can be categorized as POPs including industrial chemicals (such as polychlorinated biphenyls/PCBs, synthetic dyes [2]), agriculture waste product (pesticide [3] and herbicide [4]), endocrine disrupting chemicals/EDCs (such as phenols [5], personal care, and pharmaceutical products/PCPPs (such as antibiotic [2]). These pollutants have become a significant global concern since many of them are carcinogenic, resistant to conventional degradation treatment, and can be bioaccumulated in species through the food chain [2]. Thus, a tremendous effort is needed to find the best wastewater treatment for elimination of POPs from water resources.

**Figure 1:**
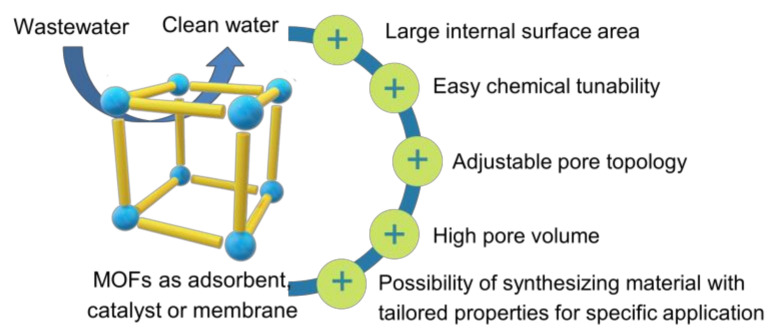
Illustration of MOFs’ advantages as adsorbent, catalyst, or membrane for wastewater treatment.

Various methods have been extensively employed for the POPs removal in wastewater such as flocculation/coagulation [6], adsorption [5,7], photocatalytic [8,9], Fenton-like catalysts [10,11], membrane separation [12,13], and reverse osmosis [14]. Among these methods, adsorption, catalytic degradation, and membrane separation are promising techniques for eliminating harmful pollutants from wastewater due to their low cost and simple process [13,15,16]. The correct choice of materials has a significant influence on the removal performance of those methods [17,18,19]. In this context, metal-organic frameworks (MOFs) have attracted tremendous attention as futuristic materials for wastewater treatment as an adsorbent, catalyst or membrane due to their advantageous features including adjustable pore topology, large internal surface area, and easy chemical tunability (Figure 1) [20,21,22].

**Figure 2:**
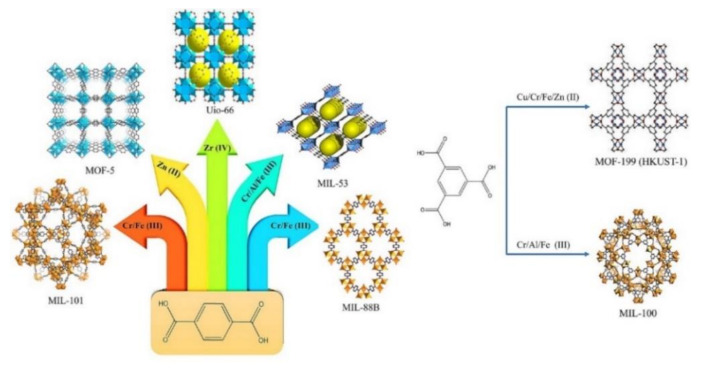
Example of unlimited combination between various metal nodes with: (**left**) 1,4-benzene dicarboxylate (terephthalic acid) to create UiO-66, MIL-101, MIL-53, MIL-88B, MOF-5, and (**right**) 1,3,5-benzene tricarboxylate (trimesic acid) to produce MIL-100 and MOF-199 (HKUST-1). Reproduced with permission from ref [19] Copyright (2021) Elsevier.

MOFs are a new class of crystalline porous materials consisting of a regular arrangement of organic and inorganic components in a rigid periodic network structure [23]. The inorganic positively charged metal ions form nodes as secondary building units that bind the organic linkers together to form a repeating cage-like structure with a hollow structure, allowing MOFs with ultrahigh porosity (up to 90% free volume [24]) and extraordinarily large internal surface area (up to 7000 m^2^/g experimentally and 14,600 m^2^/g hypothetically [25]). Thanks to the nearly unlimited combination between metal nodes and organic linkers as their building blocks, MOFs enjoy an exponential growth with more than 90,000 structures found to date [22]. An example of the unlimited combination of MOFs structures that can be obtained between various metal nodes and two of the most favorable linkers (terephthalic acid and trimesic acid) can be seen in Figure 2 [19].

**Figure 3:**
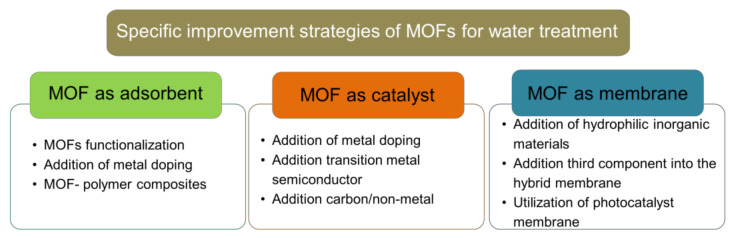
Specific improvement strategies of MOFs as adsorbent, catalyst and membrane for wastewater treatment.

Although MOFs display good performance for wastewater treatment, its application is limited by its instability in water [26]. Several MOFs such as MOF-5 and MIL-101-V undergo ligand displacement caused by hydrolysis in water exposure, which degrades the MOF [27]. Nevertheless some MOFs such as MOF-74, MIL-101 (Al, Fe, Cr), MIL-53 (Al, Cr), UiO-66, and ZIF-8 show good stability in water [28,29,30,31], making these MOFs more suitable for wastewater pollutant adsorbent application. MOFs with a comparatively strong Mg-O bond (MOF-74), Zn-N bond (ZIF-8), Cr-O bond (MIL-101(Cr)), and Al-O bond (MIL-53(Al)) between their metal ion and organic ligand have been reported to have higher water stability than MOFs with a weaker Cu-O bond (HKUST-1) and Zn-O bond (MOF-5, MOF-508) [32]. Considering that MOFs water stability is mandatory requirement for their application in wastewater treatment, recent approach to enhance MOF’s water stability is discussed as a general strategy in this review. Furthermore, this review highlights recent strategies to improve water-stable MOFs’ performance that correlated with their specific role as an adsorbent, catalyst, or membrane. These include a novel solution either by MOFs functionalization, addition of metal doping, transition metal, semiconductor, or non-metal addition. A summary of specific improvement strategies can be seen in Figure 3. To date, discussion on many reviews only focuses on one or two specific roles of MOFs in wastewater treatment. Thus, this review might hold an advantage since this review offers an up-to-date strategy (from 2015 to 2021) with broader perspectives to cover all MOFs’ roles in wastewater treatment, either as an adsorbent, catalyst, or membrane.

## 2. General Improvement Strategies: Enhancing the Water Stability of Metal-Organic Frameworks

For application in wastewater remediation where contact with water molecules is unavoidable, water stability is a must-have prerequisite in order for MOFs to be viable for use. Early MOFs such as MOF-5 are reported to be unstable in water (or steam/vapor), as the Zn^2+^ ion is not a high-valence ion that can support strong Zn-O coordination bond [33]. Recent advancements have led to the creation of thermodynamically water-stable MOFs such as the UiO-series and ZIF-series MOFs [34]. In general, the water stability of these MOFs can be attributed to its metal-ligand coordination bond, which is stronger than the bond between the MOF’s metal center and water molecules, thus preventing water from taking over and hydrolyzing the bond. This can be achieved by using high-valence metal ions (e.g., Zr, Ce) instead of lower valence ions (e.g., Zn, Cu) [35]. While the aforementioned criteria are necessary to enhance the MOF’s thermodynamic stability, it is not the sole governing factor of the MOF’s stability in water. Another aspect to consider is kinetic stability, in which stability can be achieved by relying on sufficiently high activation energy barrier (*E_a_*) being present [36]. Thus, even if a MOF does not possess inherently good thermodynamic stability (i.e., water could hydrolyze the coordination bond if it manages to reach the metal core), stability may still be achieved by kinetic factors that presents activation energy barrier (i.e., the water could not even come near the metal core), including steric effects and hydrophobicity. In the former case, high metal coordination numbers can create a crowding effect, as well as the presence of ligands with aromatic structure that creates steric hindrance. In the latter case, hydrophobicity itself can be divided into two criteria: (1) hydrophobicity in the MOF’s internal structure, and (2) hydrophobicity in the surface/pores.

**Figure 4:**
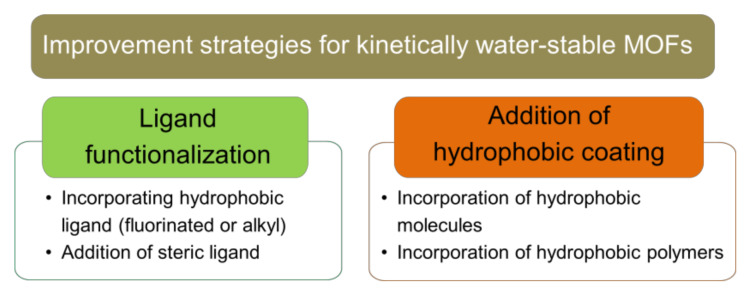
Improvement strategies for kinetically water-stable MOFs through post-synthesis modification.

As this review aims to mainly discuss the improvement strategies on existing MOFs in wastewater remediation, strategies that involves changing the internal MOF’s structure/component during synthesis (i.e., thermodynamic stability) will not be discussed. A comprehensive review and compilation discussing the relation between each of the MOF’s structural factors and its water stability has been elegantly reviewed by Burtch et al. [36]. This section of the review will mainly focus on discussing the post-synthesis modification of existing MOF structure to make it more water-stable, which includes surface hydrophobic modification, as well as ligand functionalization of existing MOFs structures and introducing hydrophobic coating. A summary of improvement strategy for kinetically water-stable MOFs is presented in Figure 4.

### 2.1. Ligand Functionalization

There are two ways in which the functionalization of ligands can improve the water stability of a given MOF. The first mechanism is by improving the internal hydrophobicity of the MOF. It has been widely reported that MOFs stability in water can be improved by incorporating hydrophobic fluorinated and alkyl functional groups on the organic ligands. The presence of a bulky and long alkyl group is demonstrated to be able to turn IRMOF-3, which is not inherently hydrophobic, into a hydrophobic material [37]. Additionally, the modification of aminated MIL-53 [MIL-53(Al)–NH_2_] by alkyl anhydrides, creating amide functional group, made the material superhydrophobic. In a separate work, attachment of fluorinated group in the form of fluorinated monocarboxylic acid, substituting monocarboxylic acid ligand in DUT-67, has been reported to improve the internal hydrophobicity and water stability of MOFs [38]. Another mechanism in which ligand functionalization can improve the water stability is by relying on steric factors. An interesting MOF to discuss here is UiO-66, where the MOF contains carboxylate ligands that have low p*K_a_*, and thus should have been susceptible to hydrolysis. The stability of UiO-66 can be attributed to two factors. The first one is due to the high coordination number, which creates a crowding effect and prevents water from clustering near the metal core. A high coordination number also means that even if one bond were to be hydrolyzed, there are still plenty of other bonding sites to support the lattice structure, therefore giving the overall structure higher tolerance before collapsing due to hydrolysis [39]. The second one can be attributed to the ligand itself, where the aromatic rings in the UiO-66 can exhibit significant rotational dynamics. DeCoste et al. demonstrated that UiO-67 which contains two aromatic rings is more susceptible to water than UiO-66 with only one aromatic ring, possibly due to the greater torsional strain created around the metal cluster [40].

Based on those principles, Zhang et al. developed a new highly water stable Zr-based MOF [41]. The group designed new Zr-based MOFs with hexacarboxylate ligands as functional groups, and the metal clusters are modified with four different functional groups, including HCOO^−^, CH_3_COO^−^, H_2_O/OH and PhCOO^−^. Addition of such functional groups can tune the MOF’s water absorption and water stability properties. In a more computational approach, Batra et al. recently developed a machine learning model to predict the water stability of MOFs, using empirically measured dataset of water stabilities from over 200 MOFs to create a relation between each of the chemical and structural features to its water stability [42]. More precisely, the model took account of the MOFs’ metal node, organic linker, and molar ratios, and the developed model has successfully predicted the water stability of several MOFs with high accuracy relative to their experimental results. This generalized model can be used as a baseline for future experiments to synthesize new MOFs with the desired level of water stability in the future.

### 2.2. Addition of Hydrophobic Coating

The other strategy to improve the water stability of an MOF is by introducing hydrophobic molecules/polymers as hydrophobic coating to increase surface hydrophobicity of the MOF. Sun et al. successfully introduced a hydrophobic octadecyl phosphonic acid (OPA) layer to the surface of several Zr-based MOFs (UiO-66, UiO-66-SO_3_H, PCN-222) through immersion in OPA ethanol solution for 24 h [43]. Addition of OPA coating on the surface Zr-based MOFs boost the MOF’s hydrophobicity from hydrophilic pristine MOFs (water contact angle (WCA) of UiO-66, UiO-66-SO_3_H and PCN-222 are 19°, 10°, and 15° respectively) to superhydrophobic OPA-coated MOFs (WCA of OPA-UiO-66, OPA-UiO-66-SO_3_H and OPA-PCN-222 are 160°, 162°, and 157° respectively). The water stability of OPA-UiO-66-SO_3_H and OPA-PCN-222 was tested by immersing the MOFs in basic aqueous solution (pH = 11) for seven days. The water stability test was conducted only in basic solution as previous study reported that the MOFs were stable in acidic solutions [44,45]. Both OPA-UiO-66-SO_3_H and OPA-PCN-222 retained their respective surface area of 1156 and 1713 m^2^/g after seven days, while under the same conditions pristine UiO-66-SO_3_H lost 53.8% of its surface area from 1156 to 534 m^2^/g and pristine PCN-222 completely dissolved into the solution.

Qian et al. treated DUT-4 with organosilicon (namely hydrophobic-treated (HT) DUT-4) by immersing the MOF in organosilicon heptane solution and found that the MOF’s WCA was significantly increased from 13 ± 2 ° (pristine DUT-4) to 148 ± 3° (HT DUT-4) [46]. Water stability of the pristine and HT DUT-4 were evaluated by immersing them in acidic, neutral, and basic aqueous solution at 50 °C for six days. The surface area of the pristine DUT-4 decreased around 80% from 1183.8 m^2^/g to 192.7, 224.5, and 206.8 m^2^/g after six days immersion in acidic, neutral, and basic solution respectively. On the other hand, HT DUT-4 retained around 80% of its initial surface area of 1125.4 m^2^/g in acidic, neutral, and basic solution after six days with surface area of 903.6, 983.8, and 935.4 m^2^/g respectively.

In addition of using hydrophobic molecules, several studies successfully increased the surface hydrophobicity of MOFs by introducing hydrophobic polymeric materials to the MOFs. Ding et al. increased hydrophobicity and water stability of HKUST-1 by creating hydrophobic layer of polydimethylsiloxane (PDMS) on the MOF surface through post-synthesis in-situ polymerization [47]. The WCA of the PDMS coated HKUST-1 (designated HKUST-1-P) and pristine HKUST-1 was 135° and 0°, respectively, which proved that the method increased the hydrophobicity of HKUST-1. HKUST-1-P showed good water stability by retaining almost completely its surface area of 1352 m^2^/g after immersion in water for three days, while the surface area of pristine HKUST-1 decreased from 1451 m^2^/g to 0.5 m^2^/g. PDMS coating method is also powerful to increase hydrophobicity of other MOFs, such as ZIF-67 and MIL-125, from the WCA of 0° (their pristine form) to 146° (PDMS-coated ZIF-67) and 141° (PDMS-coated MIL-125), respectively. In a separate work, Yi et al. used catechol-functionalized PDMS (PDMS-Cat) also showed hydrophobicity improvement of ZIF-8 from the WCA of 0° (pristine form) to 126° (PDMS-Cat coated ZIF-8) [48]. Interestingly, stronger hydrophobicity was observed when PDMS-Cat-coated ZIF-8 casted on the carbon cloth substrate (WCA of 156°) since the excess PDMS-Cat that were present in the mixture will form a network and homogeneous MOF coating on the substrate. It is noteworthy that both researchers reported that PMDS [47] or PDMS-Cat [48] coating not only boost hydrophobicity of the MOFs, but also retain their catalytic performance in the presence of water molecules.

## 3. Specific Improvement Strategies Related to Each Role of Metal-Organic Frameworks in Wastewater Treatment

### 3.1. Metal-Organic Frameworks as Adsorbents

Adsorption is an attractive method to remove pollutants from wastewater due to its simplicity and relatively low cost [49]. The most commonly used adsorbents for wastewater pollutant removal is activated carbon (AC) [50]. AC is popular due to its high surface area (up to 1100 m^2^/g) and high pore volume (up to 0.40 m^3^/g) [51], which facilitates effective adsorption of pollutants (up to 50% of initial concentration) [52,53,54]. However, the ideal adsorbents not only have high adsorption capacity, but also high adsorption selectivity of the persistent organic pollutants [34,35]. In this context, MOFs are promising alternative adsorbents due to their extraordinary high surface area (up to 7000 m^2^/g [25]), high adsorption capacity, ability to bind various organic pollutant, and adsorption selectivity [7,55,56]. Haque et al. shown that MIL-101(Cr) has higher methyl orange (MO) adsorption capacity (114.0 mg/g) than AC (11.2 mg/g), due to surface area of MIL-101(Cr) (3873 m^2^/g) is significantly higher than AC (1068 m^2^/g) [57]. MOFs also show good adsorbance of other emerging pollutants such as antibiotics and polycyclic aromatic hydrocarbon (PAH). In a study by Chen et al. [58] UiO-66 showed significantly higher adsorption capacity of tetracycline hydrochloride (TH; 23.1 mg/g) antibiotics compared to the adsorption capacity of AC for the same substance (1.98 mg/g) previously reported [59]. In a report by Zango et al. [60] MIL-88(Fe) showed an adsorption capacity of 23.6 mg/g for adsorption of highly toxic PAH anthracene (ANT), performing better than AC with an adsorption capacity of 8.35 mg/g in a previous report [61]. Several other reports of MOF application as adsorbents of wastewater emerging pollutant and their adsorptive performance are listed in Table 1. In this section, three strategies to further improve the performance of water-stable MOFs as wastewater pollutant adsorbents will be discussed. Those methods are functionalization, metal doping, and MOF-polymer composites.

#### 3.1.1. Effects of Functionalization

In most cases, MOFs adsorb organic pollutants through π‒π interactions. Thus, modifying MOFs by adding additional organic groups such as ‒NH_2_, ‒COOH, or ‒SO_3_H as functionalization could improve their adsorption capacity by providing an additional interaction through electrostatic attraction or hydrogen bonding [65,66,67,68,69,70,71,72]. Furthermore, ligand functionalization of MOFs could also enhance their pollutant adsorption selectivity, stability, and reusability.

Sulfonic acid (‒SO_3_H) functionalization of the MOF is effective to improve its adsorption capacity against anionic organic pollutants through electrostatic interaction since sulfonic acid functional groups could increase the positive charge of the MOF. Yang et al. found a significant improvement of adsorption capacity of sulfonic acid MIL-101(Cr) (MIL-101-SO_3_H) against anionic synthetic dyes, such as MO and congo red (CR) [66]. They observed that MO adsorption capacity of MIL-101-SO_3_H (688.9 mg/g) is 1.7 times higher than pristine MIL-101(Cr) (406.1 mg/g). While CR adsorption capacity of MIL-101-SO_3_H (2592.7 mg/g) is 1.9 times higher than its pristine form (1367.1 mg/g). An anomaly phenomenon was observed on the adsorption capability of MIL-101-SO_3_H against acid chrome blue K (ACK) dyes. Even though ACK is also a negatively charged dyes molecule, the nonlinear structure of ACK molecules hinders π‒π interactions between the MOFs and the dye. As a consequence, the ACK adsorption capacity of MIL-101-SO_3_H (213.2 mg/g) is 0.7 times lower than its pristine form (323.1 mg/g). It is noteworthy that MIL-101-SO_3_H showed good reusability characteristic with less than 12% decrease of capacity after five cycles of adsorption and desorption process.

**Figure 5:**
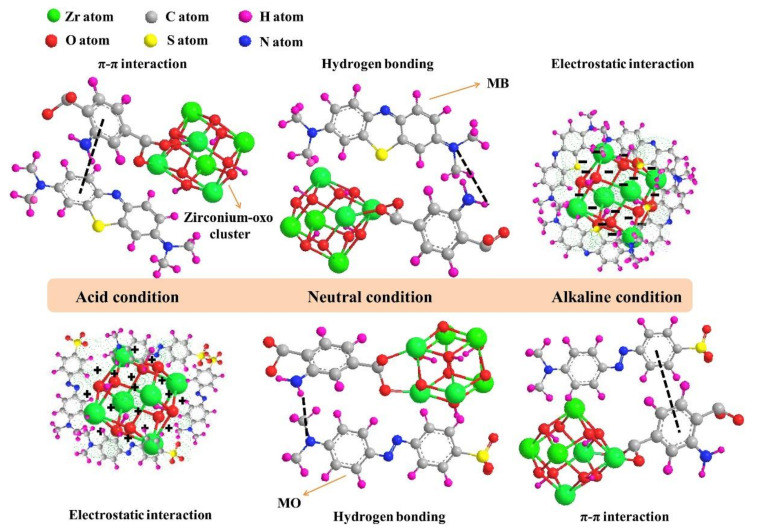
Schematic illustration of adsorption mechanism of the aminated MOF against anionic MO and cationic MB at various pH. Reproduced with permission from ref [65] Copyright (2019) Elsevier.

Amination (addition of amine functional group) is one of MOFs’ functionalization strategy and demonstrates fascinating features. Not only it could enhance MOFs adsorption capacity by providing an extra interaction through hydrogen bonding and/or electrostatic interaction, but also it could control MOFs adsorption capacity and selectivity by tuning their pH [65,67,68,69,70,71]. Lv et al. showed that the introduction of amine could increase the adsorption capacity of UiO-66 by around 150% and adsorption capacity and selectivity of the aminated MOF (UiO-66-NH_2_) is strongly affected by pH [65]. At acidic condition, UiO-66-NH_2_ tend to adsorb anionic MO through electrostatic interaction, because the positive surface charge of the MOF was formed in acidic condition as the result of amine protonation. On the contrary, adsorption of cationic MB is preferable at basic conditions through electrostatic interaction due to the presence of the negatively charged UiO-66-NH_2_. Interestingly, amination could also enhance MOFs adsorption capacity against both MO and MB at neutral condition by providing additional hydrogen bond between the MOF and the dyes. Schematic illustration of the adsorption mechanism of the aminated MOF against anionic MO and cationic MB at various pH can be seen in Figure 5.

Zhuang et al. used similar aminated MOF (UiO-66-NH_2_) for anionic diclofenac (DCF) adsorption and they found that the DCF adsorption capacity of the aminated MOF (555 mg/g) is 1.5 times higher than its pristine MOF (357 mg/g) [71]. Amine groups in UiO-66-NH_2_ increased its adsorption capacity by acting as DCF adsorption sites through hydrogen bonding and positively charged the aminated MOFs, which are favorable for anionic DCF adsorption through electrostatic interaction.

The effect of amination on elevating adsorption capacity of MOF are also observed on other MOFs and different organic pollutants. Park et al. reported that amination on MIL-101(Cr) lead to 260% improvement on bisphenol S (BPS) adsorption capacity, from 196 mg/g (pristine MOF) to 513 mg/g (aminated MOFs) because of the presence of hydrogen bonding between BPS and amine group [68]. Yu et al. used the aminated MIL-53(Fe) for tetracycline (TC) adsorption and discovered that TC adsorption capacity of the MOF was increased from 248.3 mg/g (pristine form) to 271.8 mg/g (aminated form) [69]. Abdelhameed et al. also found that amination is effective to improve the dimethoate adsorption capacity of MIL-53(Al) [67]. Interestingly, they revealed that its adsorption capacity could increase even further by mixing terephthalic acid (H_2_BDC) and 2-aminoterephtalic acid (H_2_BDC-NH_2_) with 1:1 molar ratio in the MOF synthesis process, designated as Al-(BDC)_0.5_(BDC-NH_2_)_0.5_. Dimethoate adsorption capacity of Al-(BDC)_0.5_(BDC-NH_2_)_0.5_ is 513.4 mg/g, which is 3.3 times higher than Al-BDC (154.8 mg/g) and 1.9 times higher than Al-BDC-NH_2_ (266.9 mg/g) [67].

An incredible improvement adsorption capacity of the MOFs against cationic pollutants could be attained by carboxyl functionalization. Gao et al. presented that the carboxyl functionalized UiO-66, UiO-66-(COOH)_2_, could achieve ultra-high adsorption of rhodamine B (RhB) with the maximum adsorption capacity of 2200 mg/g at high RhB concentration, which is 11 times higher than its pristine form (200.4 mg/g) [72]. Carboxyl functionalization increased the RhB adsorption of UiO-66 by altering the surface charge of the MOF. The surface of UiO-66 is positively charged in acidic pH, while the surface of UiO-66-(COOH)_2_ is strongly negatively charged at the same range, enhancing the adsorption of the MOF for cationic pollutants such as RhB.

Other MOFs functionalization such as -OH, -NO_2_, and -Br could also enhance MOFs adsorption capacity. Hydroxyl functionalization could improve the adsorption capacity of MIL-101(Cr) against various organic pollutants by providing an additional hydrogen bonding between -OH group and organic pollutant [70]. While -NO_2_, and -Br functionalization could enrich TC adsorption capacity of MIL-53(Fe) by increasing the positive charge of MIL-53(Fe) that lead to improvement of electrostatic attraction between the MOF and TC [69]. The effect of functionalization on adsorption capacity of the MOF are summarized in Table 2.

#### 3.1.2. The Influences of Metal Doping

The addition of metal atoms may affect the charge of the MOFs and subsequently enhance the electrostatic attraction between the MOFs and pollutants. In another report by Yang et al. the effect of Ce addition on the dye adsorption capabilities of UiO-66 for MB, MO, CR, and ACK. Ce-loaded UiO-66 has a larger surface area of 1135 m^2^/g compared to pristine UiO-66 with surface area of 981 m^2^/g [73]. The Ce-loaded UiO-66 shows MB, MO, CR, and ACK have an adsorption capacity of 145.3, 639.7, 826.7, and 245.8 mg/g respectively, higher than pristine UiO-66 with the capacity of 24.5, 172.5, 495.0, and 230.9 mg/g in the same order. The increase of MB adsorption in the Ce-loaded UiO-66 is due to the decrease of positive charge following Ce addition, which reduces the repulsion between the MOF and the cationic MB. Increase of anionic dyes MO and CR adsorption, despite the decrease of positive surface charge of the MOF, is due to Ce particles serving as additional active sites of the MOF, which adsorb the dyes through π‒π interactions. The adsorption of ACK did not increase in Ce-loaded UiO-66, presumably due to the non-linear structure of ACK molecules which hindered π‒π interactions between the ACK dye and Ce sites, preventing additional dye adsorption by the Ce particles.

Zhang et al. reported the effect of Fe-doping on the adsorption capability of Zr-based MOF of MB and MO [74]. The Fe-doped MOF-545, or Fe-loaded MOF-545(Fe) shows lower adsorption of MB and higher adsorption of MO compared to the undoped MOF-545. The Fe-loaded MOF-545(Fe) has 382.35 and 803.664 mg/g of MB and MO adsorption capacity respectively, while the undoped MOF-545 has 906 and 589 mg/g of MB and MO capacity respectively. The decrease of MB adsorption capacity is due to the increase of the positive surface charge of the MOF following Fe doping, which also results in better attraction and adsorption of MO.

Yang et al. reported the effect of Mn doping to the adsorption capabilities of UiO-66 for tetracycline (TC) adsorption [75]. The maximum adsorption capacity of the Mn-doped UiO-66 (Mn-UiO-66) for TC was 184.49 mg/g, higher than the maximum TC adsorption capacity of pristine UiO-66 in previous reports which was 23.1 mg/g. Mn-doping increased the amount of adsorption active sites on the MOF through donation of valence electrons, increasing the adsorption capacity of the MOF. The Mn-UiO-66 also showed good reusability, retaining 84% of its initial adsorption capacity after three cycles. Sun et al. [43] reported the effect of Cu doping to the adsorption capabilities of ZIF-8 for tetracycline (TC) adsorption. The reported maximum TC adsorption capacity of the Cu-doped ZIF-8 (Cu-ZIF-8) was 307.9 mg/g, 2.4 times higher than the adsorption capacity of pristine ZIF-8. The Cu-doping increased the adsorption capacity of ZIF-8 by donating valence electrons to the MOF. Cu-ZIF-8 also showed very good stability in water and reusability. The adsorption capacity of Cu-ZIF-8 sample used for the reusability test only decreased to 139.8 mg/g from the initial capacity of 156.5 mg/g after four cycles.

#### 3.1.3. MOF-Polymer Composites

MOF can be combined with polymers to create MOF-polymer composites. This way, the MOF’s porosity can be enhanced, new functionalities can be imbued, and its stability can be improved [76]. The composite can be formed by in situ polymerization inside the MOF’s pores, constructed either by polymeric ligands, introduced post-synthesis (by covalent grafting or incorporating separately made polymer), or self-assembly of MOFs around pre-synthesized polymers. Rather than enhancing the adsorptive capability of the MOF, the creation of MOF-polymer composites often aims to ease the process of recovery and separation, as well as improving the stability of the MOF itself. However, performance is not always compromised, and new reports often aim to reach a fine balance between performance and practicality.

Several MOFs are known to exhibit poor stability in aqueous media due to the presence of species which can hydrolyze the metal or protonate the ligands (e.g., H_2_O, H^+^, OH^−^). This can be largely remediated by grafting polymers onto the MOF’s surface, as was demonstrated by Hou et al. [77]. The group grafted polymethylmethacrylate (PMMA) onto UiO-66-NH_2_ by soaking the MOF into solvent containing MMA monomers, followed by UV irradiation, forming polymer brushes on the surface of the MOF. The presence of the polymer brushes were reported to be able to enhance the MOF’s chemical and thermal stability, without significantly decreasing the porosity and surface area for adsorption. As with many reports with MOF-polymer composite, decrease in BET surface area is expected as a compromise for significantly enhanced stability. Nevertheless, the presence of flexible grafted polymers on the MOF’s surface may help to compensate with the loss in surface area in terms of adsorption performance by filling the gaps between the MOF’s particle and promote pollutant transport via the MOF’s pores. As such, the presence of grafted polymer in this report were able to enhance the removal efficiency of R-250 dye despite the loss in surface area.

To enhance reusability and facilitate separation after usage, Abdi and Abedini prepared polyether sulfone (PES), alongside ZIF-8/ZIF-67 nanocomposite in the form of beads, using a one-step phase inversion method [78]. The addition of MOF significantly improves the polymer’s BET surface area and total pore volume, even though it is still lower than pristine ZIF-8 and ZIF-67. The nanocomposite shows low adsorption capacity at extremely low pH due to the repulsive electrostatic interaction between ZIF and malachite green, but gradually improves when the pH is increased due to π-π stacking interaction. In ideal conditions, the composite shows significantly higher removal capacity compared to bare PES, but is still slightly lower than the two pristine ZIFs. However, the goal to enhance reusability was achieved, with the composite displaying nearly similar performance as freshly prepared sample even after six repeated uses.

An ice templating method has been used by Fu et al. as an alternative to the commonly employed freeze-drying method in assisting the fabrication of chitosan/UiO-66 composite [79]. Instead of the usual powder form commonly found in MOF-based reports, the composite was made in monolith form so that it can be easily picked up using tweezers for separation and subsequent use. The monolithic structure was achieved by blending the MOF with polymer solution and allowing the solvent to evaporate. Chitosan was chosen as it is naturally abundant, and has been proven to exhibit good adsorptive capability against various anionic and cationic dyes [80]. It was reported that ice templating creates a porous material with highly interconnected porosity, and exhibit adsorption capacity close to that of pure UiO-66. Moreover, it is easy to separate and can retain most of its adsorptive capability over subsequent cycles, thus ensuring practicality over the conventional powder-based adsorbent.

### 3.2. Metal-Organic Frameworks as Catalyst in Catalytic Degradation of Wastewater Pollutant

As previously discussed, the MOFs’ general advantages as a catalyst means that the material has a huge potential to be a better catalyst relative to conventional photocatalysts, as well as conventional Fenton or Fenton-like catalysts. Nevertheless, improvements must be made to address its current weaknesses and make them viable for practical applications. Recently, mixed-metal MOF systems, which are MOFs comprising of two or more metal ions as nodes in the same MOF phase, have demonstrated excellent results in MOF-based catalysis [81]. This is usually achieved by doping metal ions into existing MOF, and the strategy will be discussed in more detail below.

Alternatively, combining two materials to create a composite system has been receiving major attention in catalysis. In this scenario, one material will act as a structural support for anchoring the catalytically active second material in order to enhance its stability and ensuring reusability [82]. Huge efforts to combine MOFs with other functional materials in order to enhance its catalytic properties have been attempted, which can be confined inside the MOFs’ structure thanks to its permanent porosity [83,84]. Among these, the addition of other catalytically active materials as guest species have been demonstrated, and the combinations are generally reported to be synergistic and successful in increasing the MOFs catalytic activity to degrade various organic pollutants found in wastewater. The combination of MOFs and other nanostructured materials including metal nanoparticles (MNPs), semiconductors, and nanostructured carbon (e.g., graphene, carbon nanotube) is an interesting topic in the field of catalysis in recent years. Such nanostructures are known to possess high surface energy. Therefore, they are prone to aggregation, which will lead to a decrease in catalytic performance. On the other hand, being a porous structure, MOFs have the potential to facilitate other catalytically active guest species to enhance its overall catalytic performance. When combined, the MOFs act as a spatial confinement to the guest species, preventing them from aggregating with each other, while still allowing for substrate transport. The catalytically active guest species, in turn, will elevate the MOFs’ catalytic activity—which is often meager on its own—by creating a composite alongside the MOF. The MOFs may act as a mere host for the guest species, or it can also participate in the catalysis process as a co-catalyst.

#### 3.2.1. MOFs as Photocatalyst

Some MOFs have been reported to display the electronic properties that are similar to a traditional semiconductor materials, thus enabling the MOFs to act as a photocatalyst by generating free radicals when illuminated by a certain wavelength of light [9]. The phenomenon was first observed by Alvaro et al. in 2007, where the group discovered that MOF-5 (consisting of Zn_4_O clusters at the corners, each connected orthogonally to six terephthalate linkers) displays behavior similar to a semiconductor upon light excitation [85]. The Zn_4_O clusters can be viewed as semiconductor dots, and the organic ligands (terephthalate linkers) can absorb light to bring the dots into its excited state, in which the photo-generated electrons are then transferred to the Zn^2+^ through ligand-to-metal charge transfer (LMCT). Since then, many other MOFs with similar working principles have been synthesized to specifically function as a photocatalyst, with examples such as UiO-66 [86] and MIL-125 [87] being the most commonly found. However, many pristine MOFs (including the aforementioned MOF-5) tend to have modest hydrolytic stability, making its application in wastewater remediation challenging. Other more stable MOFs often have large energy band gap, causing them to have modest photocatalytic activity instead. This section of this review will highlight the basic concepts of enhancement via doping and/or creation of nanocomposites with metal, semiconductors, and carbon-based materials, as well as providing examples of recent advances in MOFs-based photocatalyst in wastewater remediation.

##### Influence of Metal Addition

Metals can be used as a dopant to create mixed-metal MOF systems or in the form of separate MNPs to create a nanocomposite with the MOFs. This holds true for both photocatalysis and Fenton-like catalysis alike. An example of metal as dopant was demonstrated by Avilés et al., who reported the addition of Zr doping onto NH_2_-MIL-125(Ti) with a specific molar ratio, aimed for degradation of acetaminophen under solar light irradiation [88]. Zr doping is known to be able to increase the photocatalytic activity of TiO_2_ due to the lower energy band gap value, while NH_2_-MIL-125(Ti) is chosen due to its large surface area and satisfactory stability. The addition of Zr is meant to substitute some of the Ti content in the MOF, and indeed changes in the MOF’s crystal structure were observed by varying the Ti:Zr molar ratio. Nevertheless, when the Zr concentrations are too high, the MOF structure were reported to be amorphous, leading to reduction in catalytic activity. In a similar manner, doping of Cu onto NH_2_-MIL-125(Ti) was also reported to enhance the MOF’s photocatalytic activity [89]. Cu was chosen as it is more electronegative than Ti, while having similar ionic radius, thus resulting in good doping efficiency. In agreement with the previous report, excessive doping of Cu leads to decrease in photocatalytic activity due to formation of defects, which may function as recombination centers for the photogenerated carriers. Even though metal doping may not necessarily enhance the surface area of the MOFs (or in this case, even slightly reduced), increase in photocatalytic activity is often achieved through other means; it is usually attributed to more efficient charge separation and charge transfer efficiency or heightened light absorption ability.

Another example was recently demonstrated by Wang et al., who doped Fe(III) onto NH_2_-MIL-68(In) for visible-light-driven photocatalytic degradation of Cr(VI) and tetracycline hydrochloride (TC-HCl) [90]. Notably, the group had previously synthesized Ag NPs loaded onto NH_2_-UiO-66 and reported significant improvement in performance, but aggregation of Ag NPs are also reported to some extent [91]. Therefore, the group opted to create an active metal center by doping Fe onto the In instead, creating NH_2_-MIL-68(InαFe1-α). Compared to the monometallic NH_2_-MIL-68(In) catalyst, the bimetallic NH_2_-MIL-68(InαFe1-α) showed improved photocatalytic activity due to the Fe(III) providing charge carrier transfer route via the metal-to-metal charge transfer process, thus reducing the electron-hole recombination, facilitating charge separation and transfering efficiency.

As evident in the previous demonstration, simply anchoring MNPs onto the MOF’s surface may not be sufficient in preventing the nanoparticle’s aggregation [91]. In order to prevent aggregation and to further boost the efficiency of noble metal catalyst for the case of MNPs/MOFs composite, many contemporary demonstrations reported various strategies to improve its activity including by creating unique architectural nanostructure such as core-shell/yolk-shell structure, or combining the MNPs with other materials such as metal oxides or carbon-based materials. Both of these were elegantly demonstrated by Tilgner et al., who reported the fabrication of MIL-101(Cr) core with Au/TiO_2_ anatase shell as visible-light-driven photocatalyst against rhodamine B as model dye [92]. Addition of precious metals (e.g., Au, Pd, Pt) into MOFs are known to be able to inhibit the photogenerated electron-hole recombination due to the formation of Schottky barrier at the junction between MOFs and the metal, leading to more efficient charge carriers separation of the catalyst system. Thus, they will practically translate into better photocatalytic performance [93]. The MOFs was used as a directing structure for the core (Au/TiO_2_) synthesis, and later to imbue stability during usage. Plasmonic Au NPs were deposited onto TiO_2_ surface, mainly to enhance visible light-harvesting ability, due to them possessing localized surface plasmon resonance effect. It was proven that the addition of Au NPs significantly enhances the photocatalyst’s performance, offering six times higher degree of conversion than MIL-101(Cr) with only TiO_2_ core. Creation of a core/shell structure also enhances the overall performance when compared to the catalyst with Au/TiO_2_ uniformly distributed across the MIL-101(Cr), which also has around six times lower degree of conversion.

##### Influence of Transition Metal Semiconductor Addition

Transition metal semiconductors are traditionally known as a material for photocatalysis, as it has a strong quantum-size effect and exhibits high photocatalytic activity. However, conventional semiconductors such as TiO_2_ suffers from large energy band gap (3.2 eV), while semiconductors with lower energy band gaps often possess poor stability when used alone. Recent strategy involves the utilization of water-stable MOFs in conjunction with nanostructured semiconductors. In this scenario, the MOFs mostly acted as a porous matrix, with the semiconductors (being the more photoactive material of the two) doing the heavy-lifting in photocatalysis by filling the porous cavities of the MOFs. In addition, coupling of low band gap MOFs are also known to reduce the semiconductor’s energy band gap, making them more applicable in visible light or solar irradiation.

To resolve the problem of TiO_2’_s large band gap, Abdelhameed et al. used Ag_3_PO_4_ NPs (a well-known narrow band gap semiconductor (Eg = 2.45 eV)) alongside NH_2_-MIL-125(Ti), creating a composite system of Ag_3_PO_4_@NH_2_-MIL-125(Ti) [94]. Although it has a narrow band gap which makes it suitable for visible-light application, Ag_3_PO_4_ suffers from fast charge recombination, is prone to undergo self-reduction to Ag, and has CB higher than O_2’_s reduction potential. In this demonstration, the Ag_3_PO_4_ NPs are equally dispersed and encircled the MOF structure, creating a unique structure known as a core-shell structure, leading to the formation of well-matched heterojunctions. The introduction of Ag_3_PO_4_ could reduce the band gap of NH_2_-MIL-125(Ti). Combined with the fact that NH_2_-MIL-125(Ti) will enhance Ag_3_PO_4’_s adsorption surface area, significant enhancement in dye degradations were observed compared to bare Ag_3_PO_4_ or pristine NH_2_-MIL-125(Ti). Ag_3_PO_4_ was once again used by Sofi et al., this time alongside plasmonic Ag NPs to enhance electron-hole separation at the composite’s interface, creating a nanocomposite system of Ag/Ag_3_PO_4_@HKUST-1 [95]. Due to it being a narrow band gap semiconductor, Ag_3_PO_4_ were used to extend the light absorption range of the system into the visible light region, in which the HKUST-1 were unsuited for as it has a wide band gap of >3 eV.

Aside from transition metal oxide-based semiconductors, sulfide-based semiconductors have also gained a surge in popularity (as narrow band gap semiconductors) as an alternative option for visible-light-driven semiconductor. However, currently available visible light-responsive semiconductors (e.g., CdS, In_2_S_3_, etc.) are prone to photo-corrosion due to sulfide ions oxidation by the photogenerated hole [96]. With a band gap of 2.2–2.4 eV, CdS has the potential to become an attractive option for photocatalysis, but is still hindered by its high electron-hole recombination rate and tendency to aggregate. Hu et al. attempted to solve this problem by combining CdS with MIL-53(Fe) (energy band gap of 2.88 eV, making it responsible to visible light) [97]. The facile pathway for photoinduced electron provided by the MOFs is believed to be able to reduce the rate of electron-hole recombination. It was shown that the addition of CdS in increasing amounts will lead to better photocatalytic activity due to larger interface. Nevertheless, the excessive amount of CdS loading may cover the active sites of the MOF, thus leading to diminishing photocatalytic activity. However, the composite still retains the problem of Cd being leached out after each use, leading to somewhat unsatisfactory reusability.

##### Influence of Carbon/Non-Metal Addition

Carbon-based materials can be tailored to exhibit extraordinarily high specific surface area (e.g., activated carbon, graphene, CNT), which can help in providing the surface area needed for pollutant adsorption. Moreover, they are known to be chemically stable, and some carbon structures are noted to possess excellent electrical conductivity due to the presence of free π-electron and low crystalline defect density. The usage of carbon alongside MOFs extends not only in nanostructured carbon, but also to non-metal carbon-based semiconductors such as carbon nitride (C_3_N_4_). Addition of carbonaceous materials were done mainly to increase the available area of the active catalytic sites and sensitization for visible-light responsive photocatalyst.

**Figure 6:**
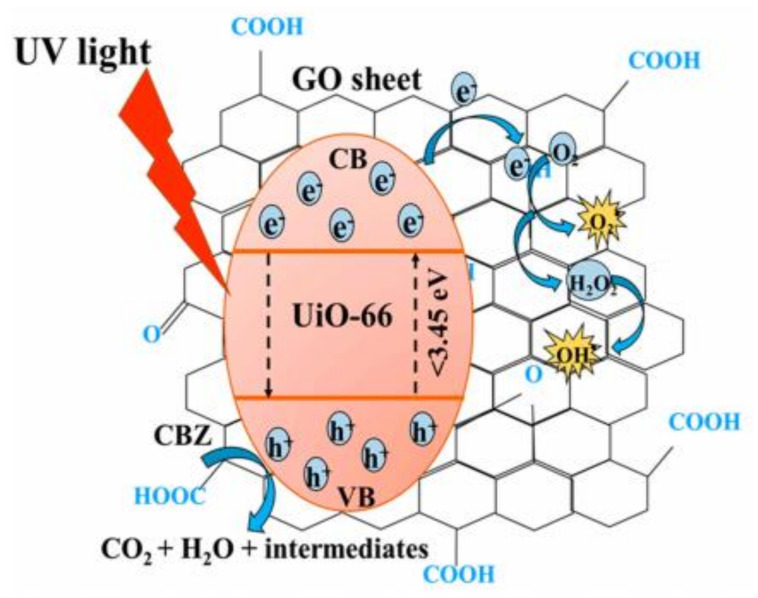
Schematic diagram highlighting the synergistical mechanism behind GO@UiO-66 nanocomposite, with GO as adsorbent, electron acceptor and band gap reductor to UiO-66, enhancing its efficiency as the active component in catalysis [98], Copyright (2020) MDPI.

Graphene and its derivative materials including graphene oxide (GO) and reduced graphene oxide (rGO) are known to be able to enhance the photocatalytic activity of the host MOF not only by increasing the adsorption surface area, but also by reducing the energy band gap, reducing electron-hole recombination, and by acting as light sensitizer. The presence of oxygen-containing functional group on the surface of GO could act as adsorption and active sites for catalysis. Heu et al. combined GO alongside UiO-66 (a zirconium-based MOF with notably excellent thermal and chemical stability) creating GO@UiO-66 nanocomposite, and investigated its efficiency in degrading carbamazepine in the UV-visible region [98]. The composite can perform in a wide range of pH, displaying good stability in both extreme acidic (pH 2) and basic (pH 10) environment. The highest photodegradation was achieved in the acidic pH range of pH 4–6, as the CBZ (pKa = 13.9) would be positively charged and the GO@UiO-66 would be in its point of zero charge, thus eliminating the effect of electrostatic repulsion due to same charge interaction. The organic ligands were excited under irradiation, and the photoelectron were transferred into the Zr-O cluster. The photogenerated electron can quickly migrate to the GO, resulting in efficient carrier separation, and the electron will react with dissolved oxygen to form the necessary radical species to degrade the CBZ who were adsorbed on the GO’s benzene structure. The synergistic mechanism between GO and UiO-66 is illustrated in Figure 6.

Another promising carbon nanostructure, carbon nanotube (CNT) was utilized by Oveisi et al. to create CNT@MIL-125(Ti) composite for dye degradation, with BDC (1,4-benzenedicarboxylate) as the organic linker [99]. Pollutant transfer occurs due to the difference in concentration between CNT and the MOF. The CNT mainly acts as photosensitizer and pollutant adsorbent, followed by efficient contaminant transfer (thanks to its tube nanostructure) into the MOF surface where the catalytic reaction occurs by the generated radical species.

Recently, *Z*-scheme heterojunction photocatalyst have received rising attention due to its potential in reducing the electron-hole recombination. Generally, *Z*-scheme can be realized by creating a ternary composite system, each with suitable energy level to bridge with one another. Recently, Cui et al. demonstrated the fabrication of a ternary composite g-C_3_N_4_/α-Bi_2_O_3_/MIL-53(Fe) as a visible light driven photocatalyst with *Z*-scheme mechanism [100]. g-C_3_N_4_ (graphitic carbon nitride) is a carbon-based nonmetal semiconductor that can be used as a substitute to the usual transition metal oxide-based semiconductors. However, g-C_3_N_4_ often shows limited photocatalytic activity due to the fast charge carrier recombination. Thus, *Z*-scheme was used to enhance this material’s activity, alongside Bi_2_O_3_, which has matching energy levels with g-C_3_N_4_ and are responsible to visible light, and MIL-53(Fe), which is a highly porous material with large surface area and wide visible light response. Due to the enhanced visible light absorption, and the construction of interfacial heterojunction which could facilitate the separation and transfer of the photogenerated charge carriers, the ternary composite displays superior photocatalytic performance relative to the single component and g-C_3_N_4_/α-Bi_2_O_3_ binary composite. Improvement strategies of MOFs as photocatalyst for wastewater treatment are summarized in Table 3.

#### 3.2.2. MOFs as Fenton and Fenton-like Catalyst

MOFs have also been extensively used as a Fenton and Fenton-like catalyst along with H_2_O_2_ to generate hydroxide radicals (•OH) [101]. Classical homogeneous Fenton catalysts features the decomposition of H_2_O_2_ with the assistance of Fe^II^ ions to generate •OH radicals. However, such catalyst systems are mostly only effective at a narrow pH range, usually at the acidic side of the spectrum. Being a homogeneous catalyst, classical Fenton catalysts are often non-recoverable and produce a high amount of iron sludge (in the form of Fe^III^ ions as the reaction byproduct) [102]. Fabrication of heterogeneous Fenton catalysts have been regarded as a prospective solution in solving these problems, with one of the strategies being to fabricate Fe-based MOFs such as MIL-100 [11] and MIL-53 [103].

Although MOFs are renowned for its large adsorption surface area, currently available MOFs often have a limited number of active sites, thus hindering its application in Fenton-like catalysis. In addition, pristine Fe-based MOFs often have poor cycling efficiency of Fe^II^/Fe^III^, making long-term usage hard to realize. Fortunately, in the same spirit as the previous section in photocatalysis, a similar strategy can be employed to improve Fenton and Fenton-like catalysis of MOFs. Rather than directly using Fe-based MOFs which contains only trivalent iron species, the MOFs can be used as a support structure as a place to immobilize the Fe^II^ ion source (usually in the form of iron oxide) on its surface to prevent catalyst loss, and to ensure continuous interaction cycles between H_2_O_2_ and the metal redox pairs (Fe^II^/Fe^III^) in order to continuously generate large amounts of reactive oxygen species [104]. Recent examples of related demonstrations will be discussed in this section of the review.

##### Influence of Metal Addition

Doping of MOFs with metals to fulfill the role of photosensitizer have attracted considerable attention in recent years. Being a photosensitizer, they do not offer much help in generating electrons when light is absent. To solve this problem, Ding et al. used Mn as a dopant to MIL-88B(Fe), alongside the incorporation of long persistent phosphors (LPPs) which are luminescent materials that can store energy and re-emit them in a long-lasting manner as a photo-Fenton catalyst [105]. Therefore, the catalyst can also maintain catalysis even in dark condition for several hours after irradiation, making it more practical for real industrial application. Doping stoichiometric amounts of Mn was noted to be able to enhance the catalytic activity. However, too much Mn will weaken the H_2_O_2’_s adhesion to the MOF’s mesoporous surface, causing a decrease in the catalytic activity. By conducting a stability test to examine the leaching rate of both Mn and Fe, the group concluded that the leached and dissolved Fe ions play little in contributing in the overall catalysis as homogeneous catalyst, and most of the catalysis were indeed done by the heterogeneous Mn-doped MIL-88B(Fe), indicating both good performance and good reusability.

To further optimize the catalyst’s properties, such as to maximize the available adsorption surface area or to create a facile pathway for substrate transfer, a specific and ordered morphology may be employed. Liu et al. employed controllable synthesis method to reliably define the morphology of Fe_3_[CO(CN)_6_]_2_, a MOF belonging to the category of Fe-Co Prussian Blue Analogues (Fe-Co PBAs) [106]. The group relies on the principle that growth temperature is able to influence the secondary building units (SBUs)’s growth rate in different crystal directions. Temperature was varied from 0 to 85 °C, producing different morphology from microspheres to microcubes at the highest temperature. The microcube morphology was shown to be the most efficient in degrading bisphenol-A (BPA), able to degrade as high as 85% in an impressive time span of only 6 min, even though its surface area is not the highest. The group linked the catalyst’s excellent performance with the percentage of exposed (100) facets, which are highly favored in activating H_2_O_2_ to generate active radical species in Fenton catalysis.

Creation of metal/MOFs nanocomposite system is also a viable strategy in enhancing the performance of MOFs-based Fenton-like catalysis. An example of this was demonstrated by Liang et al. who immobilized Au, Pd and Pt nanoparticles onto the surface of MIL-100(Fe) MOF [107]. Despite the slight reduction in the overall surface area, the resulting MNPs@MIL-100(Fe) composite shows enhanced photocatalytic activity towards methyl orange (MO) and Cr(VI) under visible light relative to the pristine MIL-100(Fe). MO was mainly degraded through the •OH radical generated by H_2_O_2_ decomposition, while Cr(VI) was reduced to Cr(III) by the photogenerated electron. The deposition of noble metals was able to improve the MOF’s charge separation efficiency by acting as a reservoir for the photogenerated carrier, leading to better photocatalysis performance. Out of the three noble metals, Pt NPs (~2 nm) shows the highest performance enhancement due to enhanced light absorption intensity and having the highest photocurrent density, indicating the most efficient charge separation out of the three.

##### Influence of Transition Metal Semiconductor Addition

As of now, transition metal oxide semiconductors such as TiO_2_ remain the most utilized semiconductors in conjunction with MOFs. Even though most of them are only active in the UV region, combining them with low energy band gap MOFs (usually MOFs with modified organic linkers or metal centers) may extend their light absorption range into the visible-light region as well. Such phenomenon was demonstrated by Li et al., who managed to anchor TiO_2_ NPs onto NH_2_-MIL-88B(Fe) as visible light driven photo-Fenton catalyst for methylene blue degradation [108]. The catalyst displays heightened adsorption capacity after introduction of TiO_2_ NPs, possibly due to the increased electronegativity, thus making it more efficient in adsorbing positively charged MB dye. Consistent with other reports utilizing nanoparticles, excessive loading of TiO_2_ NPs leads to diminishing catalytic activity due to the NPs covering the MOF’s active sites. Interestingly, although moderate, the catalyst displays some activity when exposed to visible light without the presence of H_2_O_2_, and likewise, when exposed to H_2_O_2_ under dark conditions, indicated that the composite somewhat has the behavior of photocatalyst and conventional Fenton catalyst. Unsurprisingly, being a photo-Fenton catalyst, the best result was obtained when the catalyst was exposed to both H_2_O_2_ and visible light, with the catalyst being able to degrade all the MB within 2.5 h period.

The importance of structural architecture in MOF-based catalysis was recently demonstrated by Yang et al., who created a heterogeneous Fenton-like catalyst system by using MOF-5 to enwrap Fe_3_O_4_ inside the hollow framework, creating a yolk-shell structure [109]. The Fe_3_O_4_ yolk possessed magnetic and catalytic properties; while the MOF shell is tasked to protect the yolk, while at the same time enhancing the catalytic activity due to the pores facilitating molecule transfer. It is important to note that even though heterogeneous catalysts are often usable in a wide range of pH, its efficiency is still greatly affected by change in pH. For instance, this composite was able to fully degrade MB within 20 min at pH 3, while full degradation was not obtained even after 70 min at pH 7. Nevertheless, loading Fe_3_O_4_ onto MOF-5 showed superior catalytic activity at every pH when compared to another similar experiment using the Fe_3_O_4_/carbon composite [110]. This composite was noted to be particularly stable due to their yolk/shell structure, reporting negligible Fe leaching of less than 0.1 mg/L after each cycle, and still retains most of its catalytic capability after five cycles of reuse.

Most reports up to this date still opted to use well-established transition metal oxide semiconductors, such as TiO_2_, ZnO, or Fe_3_O_4_. Choosing and trying a different base material altogether might be another viable strategy to improve the MOF’s catalysis performance. Nguyen et al. compared the usage of Fe_3_O_4_ against NiFe_2_O_4_ in conjunction with MIL-53(Fe) as visible-light driven photo-Fenton catalyst against RhB [111]. The group reported that NiFe_2_O_4_@MIL-53(Fe) exhibited better catalytic performance than Fe_3_O_4_@MIL-53(Fe), which in turn are still significantly better than that of the bare Fe_3_O_4_, NiFe_2_O_4,_ and MIL-53(Fe). Unfortunately, the catalyst’s reusability, which is one of the main advantages of MOF-based heterogeneous Fenton-like catalysts, was not reported. Nevertheless, this result might motivate other research groups to further investigate the combination NiFe_2_O_4_ or other relatively unexplored semiconductors with MOFs in the future.

##### Influence of Carbon/Non-Metal Addition

The usage of carbon nanostructures such as CNT and graphene materials alongside MOFs also extends to Fenton and Fenton-like catalysis. Zhang et al. incorporated specially modified CNT onto MIL-88B-Fe, with the CNT prior modified to contain abundant electron-rich oxygen functional groups (e.g., -COOH, -OH) in order to improve Fe(II) content for enhanced Fenton-like catalysis performance [112]. An increase in Fe(II) content in proportion to the increase in CNT and oxygen-rich functional group content were confirmed by using XPS, which can be attributed to the efficient electron transfer between the functionalized CNT and the MOF. The group shows that the catalyst was highly able to completely degrade various emerging pollutants effectively in a relatively short time.

A core-shell structured ternary composite consisting of iron oxide semiconductor, GO, and an Fe-based MOF was fabricated by Gong et al. as photo-Fenton catalyst to degrade 2,4-dichlorophenol [113]. The MIL-100(Fe) acts as the shell, surrounding layers of GO sheets, which in turn surrounds the Fe_3_O_4_ innermost core, creating a system of Fe_3_O_4_@GO@MIL-100(Fe) nanocomposites. Introduction of planar GO onto the structure can effectively prevent the electron-hole recombination by acting as an electron transfer channel from the MOF onto Fe_3_O_4_. By doing so, GO promotes the cycle of Fe(III)/Fe(II), thus making the catalyst to possess good catalytic activity. Another demonstration of ternary composite was very recently reported by Bagherzadeh et al., who created a highly active magnetic ternary structure comprising of MIL-101(Fe), GO, and CoFe_2_O_4_ [114]. The system was able to effectively act as both visible light photocatalyst, as well as photo-Fenton catalyst when used in conjunction with H_2_O_2_. Nevertheless, when used against particularly persistent Azo dye (e.g., ReR-198 in the experiment), the catalyst displays much higher activity when used as photo-Fenton rather than photocatalyst alone, which is unsurprising since photo-Fenton can be seen as a coupling of both photocatalytic and Fenton reaction.

Li et al. fabricated g-C_3_N_4_/PDI@NH_2_-MIL-53(Fe) by in situ growth of the MOF onto the g-C_3_N_4_/PDI (pyromellitic diimide) layer [115]. When added to g-C_3_N_4_, PDI can tailor the electric band structure between g-C_3_N_4_ and NH_2_-MIL-53(Fe) to better match with each other. The presence of such matched energy heterojunctions allows for more efficient electron-hole separation, as the electron from g-C_3_N_4_/PDI’s CB can be transferred easily to the MOF’s CB and, conversely, the holes in the MOF’s VB can also easily transferred to the g-C_3_N_4_/PDI’s VB. As such, efficient photo-Fenton catalytic activity can be earned due to the formation of heterojunctions caused by the excellent interfacial contact and matching electric band structure. The catalyst was reported to be highly effective in degrading a wide range of pollutants, including several antibiotics (TC and CBZ), BPA, and phenols. Summary about improvement strategies of MOFs as Fenton and Fenton-like catalyst for wastewater treatment is presented in Table 4.

### 3.3. Metal-Organic Frameworks as Membrane

Membrane-based separation technology is one of the fascinating technologies to eliminate POPs from wastewater due to its high efficiency, easiness to upscale, and the fact that it does not change the phase of the material [12,116]. MOFs based membranes received a lot of attention from researchers in 2010 due to their high permeability, selectivity, and photocatalytic activity [12,116]. Generally, MOFs as single building materials of membrane are not applicable since MOFs are expensive and their high crystallinity can produce brittleness which limits their application in membrane technology [13]. Thus, MOF membranes were commonly growth on the support materials i.e., polymers or inorganic materials to reduce cost production and improves mechanical properties [117,118,119]. The other strategy that massively explored by researchers is mixing polymeric membranes with MOFs as filler to produce a hybrid membrane or mixed matrix membrane (MMM). This strategy is attractive since it offers easy preparation, controllable pores, and is reliable for mass production. In this section, we focus on these two strategies to improve MOFs-based membrane performance for water purification.

#### 3.3.1. Growing Metal-Organic Frameworks Crystals on the Support Materials

There are several methods that have been developed to prepare pure MOF membrane on the support materials, such as in situ growth, seed assisted or secondary growth, and the electrochemical deposition growth method. The in situ growth method is prepared by growing MOF crystals on the support surface such as gold [117], alumina [118,120], titanium [121], and dan organic polymers [122]. This method is capable of growing MOF crystals with a thickness of 0.3–100 nm. However, inhomogeneous crystal growth on the support surface is often produced and limits the application of this method. Recently, it has been reported that UiO-66 NH_2_ MOF on polyacrylonitrile (PAN) substrates through the in situ growth method produces high water permeability 62 L/m^2^·h (LMH) and dye (Rhodamine B) rejection up to 92% [123]. With the same method, Guo and coworkers reported that UiO-66 under a wood membrane showed high capability to remove organic pollutants with efficiency up to 92% and permeability 1000 LMH [13].

Similarly, the formation of the MOF membrane on the support can be carried out by seed assisted or secondary growth. In this method, MOF membrane growth from pre attached crystal seeds and could control nucleation and crystallinity [124]. MOF membrane prepared by secondary growth method has good performance in gas separation [125,126] and pervaporation [127,128]. Xu et al. successfully growth Lac-Zn on the polytetrafluoroethylene (PTFE) surface and shows good efficiency on iodide removal [127]. The membranes have high removal efficiency 92.9% at solution pH 6 or 7, but less for higher and lower pH. On the other hand, the modified Lac-Zn membrane demonstrated high rigidity corresponding to low elasticity and mechanical properties. Another work, MOF-5, was prepared on the alfa-alumina surface [128]. The MOF-5 membrane effectively separate pure and mixture of toluene, *o*-xylene, and 1,3,5-triisopropylbenzene (TIPB). The membrane was prepared through two steps, which are seed preparation with precursor zinc nitrate hexahydrate and terephthalic acid in the dimethylformamide media. This was followed by dip-coating and secondary growth with reaction between zinc nitrate hexahydrate, terephthalic acid and N-ethyl diisopropylamine in same media. Finally, the MOF-5 membrane was activated at 100 °C for 6 h. The produced membranes exhibit higher pervaporation flux for pure component and separation factor for binary mixture up to 27.7 of toluene/TIPB.

The electrochemical deposition (EDS) method was proposed by many works to overcome inhomogeneous of MOF formation prepared by in situ and secondary growth method [129,130]. The MOF membrane generated via the EDS method could be achieved by anodic dissolution and reductive deprotonation [131]. This method possibly controls the thickness and repairs the MOFs crystal defect [13]. Other advantages of EDS method are low temperature reaction, scalability, and short reaction time [132,133]. Li et al. [134] reported ZIF-8 membrane electrochemically deposited to polyether sulfone (PES) ultrafiltration membrane with various ageing time and EDS time. The performance of produced membranes was characterized by water permeability and salt rejection. ZIF-8 modification results in increasing of surface roughness up to 22.1 ± 0.8 nm for 2 min of ageing and EDS time, compared to unmodified membrane roughness that was 2.7 ± 0.1 nm. Water permeability reduced by increasing of ageing and EDS time, but salt rejection significantly increased. At 2 min ageing and EDS time, high percentage salt rejection was obtained of 90.3% and 96.9% for MgSO_4_ and Na_2_SO_4_, respectively.

#### 3.3.2. Utilization of Metal-Organic Frameworks as Filler in Mixed Matrix Membrane (MMM)

##### Metal-Organic Frameworks as Antifouling Filler

Currently, hydrophobic polymeric-based membranes dominate membrane applications in water purification. Polymer materials such as polyethersulfone (PES) [135,136], polysulfone (PSU) [137,138], and polyvinylidene fluoride (PVDF) [139,140] are widely used due to their high mechanical properties and chemical resistance. However, the occurrence of fouling on the membrane surface is troublesome that can reduce membrane performance. To answer this problem, the addition of inorganic materials with hydrophilic characteristics in their structures such as clays [141,142], mesoporous silica [143], zeolite [144] and MOFs [145,146] to form a hybrid membrane or MMM is attractive since it will provide good antifouling properties.

MOFs are an attractive material for this purpose due to their tunable surface charge and ability to alter hydrophilicity of membrane matrix. Dehghankar et al. reported the effect of nanofiller addition (UiO-66, MIL-101 and FAU zeolite) to PVDF membrane on its surface properties, porosity, water permeability, membrane selectivity and anti-fouling properties [147]. The addition of inorganic nanofiller improves membrane porosity due to fast liquid-liquid demixing and has larger cavities than pristine PVDF membrane which confirmed from cross-section FESEM image. The produced MMMs exhibited higher surface hydrophilicity and pure water permeability. Membrane selectivity was evaluated toward Bovine Serum Albumin (BSA) solution having rejection up to 98%, 100%, and 97% for PVDF/UiO-66 (0.05%); PVDF/MIL-101 (0.1%) and PVDF/FAU (0.1%), respectively. Li and co-workers published HKUST-1 nanofiller modification to PES membrane [148]. It has been claimed that HKUST-1 modified membrane generates nanovoid in the membrane surface and increase surface porosity with increasing of nanofiller content. Introduction of poly(methyl methacrylate-co-methacrylic acid) (PMMA-co-MAA) copolymer also increased homogeneity of membrane pores and nanofiller dispersion. PMMA-co-MAA facilitated polar/nonpolar interaction between HKUST-1 and PES polymer chain. The separation performance yielded improvement of pure water fluxes up to 490 LMH and BSA rejection 96% for PES/HKUS-1 0.3 wt%.

**Figure 7:**
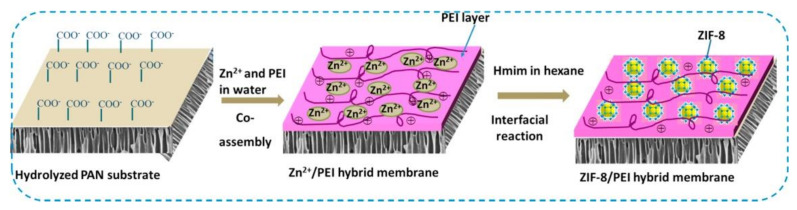
Schematic illustration of ZIF-8/PEI hybrid membrane preparation process, Reproduced with permission from ref [153] Copyright (2017) Elsevier.

Several researchers showed that MOFs-based MMM technology have high performance in dye filtration [149,150,151,152]. Mahdavi et al. used MIL-53(Al) to PSU via NIPS to produce PSU-MIL-53(Al) composite membrane [145]. The membrane was characterized to various dyes rejection such as reactive red (RR), direct yellow (DY), methyl green (MG), and crystal violet (CV). Results showed that the PSU-MIL-53(Al) membrane improved water wettability, permeability (flux), as well as dyes rejection. The highest separation performance obtained from membranes containing 0.06 wt% MIL-53(Al) with dye rejections of 99.8, 99.5, 99.2, 98.8 and 97.1% for RR, MG, DY, CV, and MB, respectively; pure water flux reached 4.8 LMH. Yang et al. prepared hybrid membrane from ZIF-8 and polyethyleneimine (PEI) on the hydrolyzed polyacrylonitrile (HPAN) substrate (Figure 7) [153]. It was found that the addition of ZIF-8/PEI to the HPAN substrate produces defect free membrane surface with selective layer 556 nm. Increasing the PEI concentration and reaction time resulted in lower water permeability, and improved methylene blue (MB) rejection. Long term filtration stability evidently proved that hybrid ZIF-8/PEI membrane stable until three-cycle filtration tests of humic acid (HA) solution and obtained flux recovery ratio (FRR) about 87.7%.

In some cases, the addition of the third component into the hybrid membrane constructed by two components was able to improve the membrane performance as proposed by Ma et al. [154]. Hybrid membranes MOF/polymers and third component such as graphene oxide (GO) have been reported by several researchers and produce high permeability and selectivity [154,155]. Makheta et al. reported Cu(tpa) MOFs supported on GO and combine with PES to produced Cu(tpa)@GO/PES composite membranes [155]. Combination GO and Cu(tpa) in PES membranes influenced surface roughness and hydrophilicity. The Cu(tpa)@GO/PES composite membranes show smoother surfaces resulting in lower interaction between contaminant and membrane surface. Membrane hydrophilicity significantly improved and affected the increase of water permeability. Lower tortuosity of composite membrane, confirmed from microscopy analysis of cross-section area, facilitated fast water permeation and also supported the water permeability data. Membrane selectivity against CR was achieved which evidently confirmed by high rejection more than 80%. Anti-fouling properties of Cu(tpa)@GO/PES composite membranes toward BSA filtration yielded improvement with FRR > 80%. In addition, composite membrane of UiO-66@GO/PES was successfully produced by Ma et al. [154]. Composite membranes were prepared via phase inversion with various concentration of UiO-66@GO composite. As a result, the UiO-66@GO/PES membrane has a smoother surface compared to PES pristine membrane. Surface hydrophilicity and water flux improved by increasing content of UiO-66@GO composite as well as antifouling properties. Dyes filtration performances toward Methyl Orange (MO) and Direct Red (DR) 80 increased with increasing of filler content and the best rejection ratio of DR 80 and MO were 98.3% and 89.0%, respectively, for 3 wt%. The present of UiO-66 could hinder stacking off GO in the polymer matrix and resulting in higher membrane selectivity.

##### MOFs as Photocatalytic Filler for Photocatalytic Membrane

Membrane fouling is initiated by particle deposition on the membrane surfaces. Membrane modification by improving the surface hydrophilicity is capable to overcome the fouling issue. However, the foulant removal on the membrane surfaces need to be achieved, one of the methods of which is through backwash. One effective method to remove foulant without backwash is by introducing a photodegradation feature. In this approach, membranes modification with photocatalyst materials, which are further known as photocatalyst membrane, is needed. As discussed in the previous Section 3.2.1, several MOFs shown good photocatalytic performance. Thus, the addition of MOFs in polymeric membrane to create photocatalytic membranes is attractive to boost membrane performance in water purification [156,157].

Zhou et al. reported self-cleaning, antibacterial, and dye selectivity properties of the MIL-125(Ti)/PVDF photocatalyst membrane [150]. The membrane was prepared by phase inversion with various concentration of MIL-125(Ti). The result revealed the optimum composition of composite membrane having water flux of 64.3 L/m^2^.h.bar and rejection of rhodamine B (RhB) of 99.7%. Photocatalytic and antibacterial properties of the membrane were examined by exposing natural light irradiation, resulting in a high flux recovery and RhB rejection by the addition of 10% of MIL-125(Ti) prior to exposure by light three cycles. The antibacterial activity indicates an inclement along with the increasing of MIL-125(Ti) concentration up to 100% with the addition of 20%. The photocatalyst reaction on the membrane is achieved due to the light induction at a certain energy level irradiating the MIL-125 which causes an electron promotion to yield a hole, hydroxyl radical, and superoxide free radical.

Li and co-workers synthesized a nanofibrous MOF membrane and measured the photocatalytic activity against methyl orange (MO) and formaldehyde (FA) [157]. This research combine polyacrylic acid (PAA), poly(vinyl alcohol) (PVA), phosphotungstic acid (PW12) and UiO-66 producing PAA-PVA/PW12@UiO-66 nanofibrous membrane by electrospinning. The cross-link occurrences of the membrane were varied between 30–120 s. In this research, a surface modification of PAA-PVA nanofibrous membrane was also conducted by growing the PW12@UiO-66 crystal via an in situ approach with the reaction time varied from 3–15 min. The result shows the high photocatalytic activity of both PW12@UiO-66 and PAA-PVA/PW12@UiO-66. The presence of FA in solution makes the degradation of MO reaching 97.35% on the addition of 3 mL FA. The cross-link reaction time influence the photodegradation of MO, indicating by the highest degradation (97.35%) achieved by 60 s of reaction time. Meanwhile, the crystal growing time on the membrane surfaces is conducted at 9 min having a degradation of MO by 97.35%. PAA-PVA/PW12@UiO-66 membrane showed good recycle performance, which have a relatively same initial water flux values at each cycle, and degradation efficiency only decrease for 4.9% after fifth cycle.

MOF-based photocatalytic membrane have also been reported in several other fields of application, such as oil and water separation [158,159], phenol removal [160], and organic pollution [47,161]; which generally show that the MOF addition within the photocatalyst membrane can significantly improve the membrane performance although still challenging to conduct. Summary of MOFs-based MMM’s performance is presented in Table 5.

## 4. Conclusions and Future Perspectives

In this review, recent advancements in the use of MOFs for wastewater treatment have been highlighted based on their character and mechanism, specifically MOFs as an adsorbent material, catalyst, and membrane. The three wastewater mechanisms are based on the superiority of the MOF properties themselves. In general, wastewater treatment using MOF material is a new breakthrough, which tries to take benefit of the superiority of MOF properties, especially from the large surface area and highly porous structure, adaptable character, and abundant active sites variations. Furthermore, MOFs can be functionalized and combined with other materials to enhance its performance or cover its weaknesses. Utilization of MOF-derived nanomaterials have shown significantly better performance in wastewater treatment against organic pollutants when compared to traditional adsorbents, catalyst, or membranes. However, it should be noted that the research results obtained are still in the laboratory testing stage.

Although MOF materials have experienced tremendous growth in various aspects, there are still limitations and challenges in implementing MOFs as a wastewater treatment material, especially in large-scale and real-life practical situations. As such, future studies in realizing practical application of MOF may consider the following points:

1. A novel synthesis method to create unique nanostructures, as well as post-treatment modification of MOFs by combining them with other materials, should be experimented further to enhance performance (e.g., adsorption surface area, charge transfer efficiency, etc.) and reusability in aqueous environment. Various uncommon materials may be tried to assess its potential synergy with the MOF (e.g., using sulfide-based instead of oxide-based semiconductors).
2. So far, most reports utilize organic dyes such as methylene blue or methylene orange. Studies should attempt to examine its effectiveness in degrading other organic pollutants (e.g., emerging pollutants such as antibiotics, pesticides, PPCPs) to determine the MOF’s effectiveness against a wide range of organic pollutants found in real-life applications.
3. The influence of pH, temperature and solute ions should be considered, as practical conditions may differ significantly from laboratory conditions. Notably, many reports only report MOF’s usage in a narrow range of pH and in a controlled aqueous medium, whereas real effluent would present more diverse pH and other ions that can affect the MOF’s adsorption and catalytic kinetic (e.g., Na^+^, Cl^−^, etc.). Development of highly adaptable MOFs that can be effective in a wide range of pH and are less influenced by other solute ions should be considered.
4. In photocatalysis and photo-Fenton catalysis, visible-light-driven catalysts are regarded as the more promising option compared to UV-driven catalysts, as sunlight comprises mostly of visible light. As such, future research may want to focus on using low band-gap MOFs, or utilizing various strategies (e.g., doping, creation of nanocomposite) to make the MOF’s band-gap more suitable for absorbing visible light.
5. Another important concern is preparing water-stable MOFs that are able to work in aqueous solution for POPs removal from the water stream. The post-synthesis approach might be crucial to prepare kinetically water-stable MOFs to retain their structure and maintain their performance in the presence of water molecules. Future research could be focused on the search of thermodynamically water-stable MOFs by discovering MOFs with a stronger coordination bond or kinetically water-stable MOFs by finding better hydrophobic coat that are suitable for MOFs.

The development of MOFs as a wastewater treatment material certainly still needs to be continued. The development is focused to further improve the performance of wastewater treatment material so that it is cheaper, practical, selective, and reusable. Many research approaches that have been taken from the direction of MOFs as adsorbents, catalysts, and membranes and have provided an understanding and conclusion that improving the performance of MOFs as wastewater treatment materials could be conducted through the functionalization of MOFs and the synergy of MOFs’ properties itself. The synergistic properties of MOF that are expected to exist simultaneously in wastewater treatment materials are properties as adsorbent, photocatalyst degradation, and separation membrane at the same time. These three properties are interrelated, and the coexistence of these three properties in a single system may be considered in designing future MOF materials for highly effective wastewater remediator. The other thing that should be focused on the future is the search for the right synthesis technique and MOF material modification, especially for preparing water-stable MOF. Another thing that needs to remain a concern in the development of this wastewater treatment material is the process of material regeneration, which should be easy and practical to make the MOF repeatedly reusable.

## Figures and Tables

**Table 1:** Examples of MOFs utilization as adsorbents for persistent organic pollutants (POPs).

MOF	Surface Area (m^2^/g)	Target Pollutant	Pollutant Concentration	Adsorption Conditions	Adsorption Capacity (mg/g)	Reusability (Cycles)	Ref.
MIL-101(Cr)	2410	Reactive Yellow 15	300 ppm	30 °C, pH 7, 24 h	397	4	[62]
		Reactive Black 5			386		
		Reactive Red 24			390		
		Reactive Blue 2			377		
	2710	2-chlorophenol	300 ppm	25 °C, 24 h	121	–	[5]
MIL-88(Fe)	1240	Anthracene	4 ppm	25 °C, pH 2–6, 25 min	23.6		[60]
UiO-66	1276	Methyl Red	200 ppm	25 °C, pH 5.5, 120 min	384	4	[63]
		Methyl Orange			454		
		Malachite Green			133		
		Methylene Blue			370		
	591.6	Tetracycline Hydrochloride	100 ppm		23.1	–	[58]
UiO-67	2345	Atrazine	6 mg/L	25 °C, 24 h	17.5	3	[64]
ZIF-8	1875	Atrazine			8.9		
CaFu-MOF	2308	Imidacloprid	0.1 mmol/L	25 °C, pH 6.5, 70 min	467.2	5	[3]

**Table 2:** The influence of functionalization on adsorption capacity of the MOF.

Functionalization	MOF	Target Pollutant	Adsorption Capacity			Reusability	Ref.
			Pristine MOF (mg/g)	Functionalized MOF (mg/g)	Improvement (%)		
**Sulfonation**	MIL-101-SO_3_H	Methyl Orange	406.1	688.9	169.6	5 cycles, 88%	[66]
		Congo Red	1367.1	2592.7	189.6		
		Acid Chrome K	323.1	213.2	66.0		
**Amination**	UiO-66-NH_2_	Methyl Orange	107.9	148.4	137.5	6 cycles, 86.2% (MO), 88.2% (MB)	[65]
		Methylene Blue	392.6	549.6	140.0		
		Diclofenac	357	555	155.5	Not Reported	[71]
	MIL-53 (Al-BDC)	Dimethoate	154.8	513.4 (Al-(BDC)_0.5_ BDC-NH_2_)_0.5_))	331.7	Not Reported	[67]
				266.9 (Al-(BDC-NH_2_))	172.4		
	MIL-101-NH_2_	Bisphenol S	196	513	261.7	5 cycles, 90%	[68]
	NH_2_-MIL-53(Fe)	Tetracycline	248.3	271.8	109.5	4 cycles, 80%	[69]
**Carboxylation**	UiO-66-(COOH)_2_	Rhodamine B	200.4	2200	1097.8	6 cycles, 73%	[72]
**Hydroxylation**	MIL-101-(OH)_3_	p-Chloro-m- Xylenol	64	79	123.4	Not Reported	[70]
		Bisphenol A	73	97	132.9		
		Triclosan	79	112	141.8		
		Ketoprofen	48	80	166.7		
		Naproxen	88	156	172.3		
**Nitro Functionalization**	NO-MIL-53(Fe)	Tetracycline	248.3	272.6	109.8	4 cycles, 80%	[69]
**Bromination**	Br-MIL-53(Fe)	Tetracycline	248.3	309.6	124.7	4 cycles, 80%	[69]

**Table 3:** Improvement strategies of MOFs as photocatalyst for wastewater treatment.

Catalyst	Experimental Conditions	Catalytic Efficiency	Stability	Ref.
NH_2_-MIL-125(TiZr_1.5_)	[ACE]_0_ = 5 mg/L [Catalyst] = 0.25 g/L pH = 6.9; T = 20 °C [Light] = 290 nm ≤ λ (simulated solar irradiation)	100% t_dark_ = 60 min t_light_ = 90 min	3 cycles >90%	[88]
Cu-NH_2_-MIL-125(Ti)	[MO]_0_ = 10 mg/L [Phenol]_0_ = 10 mg/L [Catalyst] = 0.4 g/L [Light] = 500 W, λ > 420 (visible light)	98.2% [MO]; 74.7% [Phenol] t_dark_ = 30 min t_light_ = 90 min [MO], 4 h [Phenol]	4 cycles ~100%	[89]
NH_2_-MIL-68(In_α_Fe_1-α_)	[TC-HCl]_0_ = 20 mg/L [Cr(VI)]_0_ = 20 mg/L [Catalyst] = 0.1 g/L [TC-HCl], 0.4 g/L [Cr(VI)] pH = 9 [TC-HCl], 2 [Cr(VI)]; T = room temp. [Light] = 300 W, λ > 420 nm (visible light)	72% [TC-HCl]; 99% [Cr(VI)] t_dark_ = 20 min t_light_ = 120 min	3 cycles ~100%	[90]
Core-shell Au NPs/TiO_2_@MIL-101(Cr)	[RhB]_0_ = 2 × 10^−5^ M [Catalyst] = 0.2 g/L pH = neutral; T = 20 °C [Light] = 400 W, λ > 400 nm (visible light)	59% t_dark_ = 120 min t_light_ = 360 min	5 cycles ~90%	[92]
Core-shell NH_2_-MIL-125(Ti)@Ag_3_PO_4_	[MB]_0_ = 10 ppm, 100 mL [RhB]_0_ = 10 ppm, 100 mL [Catalyst] = 0.5 g/L T = 25 °C [Light] = 100 W, 315 nm ≤ λ ≤ 1050 nm (visible light)	~100% (MB); ~90% (RhB) t_dark_ = 30 min t_light_ = 50 min (MB), 180 min (RhB)	5 cycles ~95%	[94]
CdS@MIL-53(Fe)	[RhB]_0_ = 10 mg/L [Catalyst] = 0.5 g/L [Light] = 500 W, λ > 400 nm (visible light)	92.5% t_dark_ = 30 min t_light_ = 120 min	3 cycles 44%	[97]
GO@UiO-66	[CBZ]_0_ = 5 mg/L [Catalyst] = 1 g/L pH = 7; T = 25 °C [Light] = λ ~ 254 nm (UV)	>90% t_dark_ = 60 min t_light_ = 180 min	5 cycles >60%	[98]
CNT@MIL-125(Ti)	[RB5] = 20 mg/L [Catalyst] = 0.1 g/L [Light] = UV	59% t_dark_ = 60 min t_light_ = 180 min	2 cycles ~98%	[99]
g-C_3_N_4_/α-Bi_2_O_3_/MIL-53(Fe)	[10B]_0_ = 10 mg/L; 50 mL [Catalyst] = 20 mg [Light] = 35 W (visible light)	100% t_dark_ = 60 min t_light_ = 45 min	4 cycles ~100%	[100]

**Table 4:** Improvement strategies of MOFs as Fenton and Fenton-like catalyst for wastewater treatment.

Catalyst	Experimental Conditions	Catalytic Efficiency	Stability	Ref.
Mn-MIL-88B(Fe) + LPPs	[Phenol]_0_ = 50 mg/L [Catalyst] = 0.1 g/mL V H_2_O_2_ = 1 mL [Light] = Xe irradiation (UV-visible)	96% (light); 98% (light-dark) t_dark_ = 2 min t_light_ = 30 min t_light-dark_ = 120 min	4 cycles ~100%	[104]
Microcubes Fe_3_[CO(CN)_6_]_2_	[BPA]_0_ = 20 mg/L [Catalyst] = 0.2 g/L [H_2_O_2_] = 2 mM; pH = 6; T = 25 °C	85% t = 6 min	Not reported	[105]
MNPs@MIL-100(Fe) [MNPs] = Pt, Pd, Au	[MO]_0_ = 20 mg/L [Catalyst] = 0.125 g/L V H_2_O_2_ = 40 μL; pH = 4; T = 30 °C [Light] = 300 W, 420 nm ≤ λ ≤ 760 nm (visible light)	100% t_dark_ = 50 min t_light_ = 40 min (Pt), 70 min (Pd), 100 min (Au)	4 cycles ~100%	[106]
TiO_2_@NH_2_-MIL-88B(Fe)	[MB]_0_ = 100 mg/L [Catalyst] = 0.2 g/L [H_2_O_2_] = 20 mM; pH = 7; T = ambient [Light] = 5 W, 380 nm ≤ λ ≤ 800 nm (visible light)	100% t_dark_ = 90 min t_light_ = 150 min	5 cycles >90%	[107]
Yolk-shell Fe_3_O_4_@MOF-5	[MB]_0_ = 50 mg/L [Catalyst] = 1.0 g/L [H_2_O_2_] = 30 mM; pH 4; T = 30 °C	100% t = 60 min	5 cycles 98%	[108]
NiFe_2_O_4_@MIL-53(Fe)	[RhB]_0_ = 100 mL, 3 × 10^−5^ M Catalyst = 20 mg [H_2_O_2_] = 0.01 mM; pH = 5; T = 28 °C [Light] = 40 W visible light fluorescent lamp	95.18% t_light_ = 180 min	Not reported	[110]
CNT@MIL-88B-Fe	[Phenol, 2,4-DCP, SMZ, BPA]_0_ = 25 mg/L [Catalyst] = 0.1 g/L [H_2_O_2_] = 2.5 mM; pH = 4; T = 25 °C	100% t = 10 min (Phenol) t = 30 min (2,4-DCP, SMZ, BPA)	3 cycles ~100%	[111]
Core-shell Fe_3_O_4_@GO@MIL-100(Fe)	[2,4-DCP]_0_ = 50 mg/L [Catalyst] = 0.2 g/L [H_2_O_2_] = 3 mM; pH = 5.5; T = room temp. [Light] = 500 W, 420 nm ≤ λ (visible light)	100% t_dark_ = 30 min t_light_ = 40 min	4 cycles ~95%	[112]
CoFe_2_O_4_@GO@MIL-101(Fe)	[DtR-23, ReR-198]_0_ = 100 mg/L [Catalyst] = 20 mg/L V H_2_O_2_ = 50 µL; pH = 3; T = room temp. [Light] = 100 W, visible light	99.93% (DtR-23), 99.65% (ReR-198) t_dark_ = 90 min t_light_ = 70 min	5 cycles ~90%	[113]
g-C_3_N_4_/PDI@NH_2_-MIL-53(Fe)	[TC, CBZ, BPA, PNP]_0_ = 50 mg/L [Catalyst] = 0.4 g/L [H_2_O_2_] = 10 mM; pH = 6, T = room temp. [Light] = 5 W, 380 nm ≤ λ ≤ 800 nm (visible light)	90% (TC); 78% (CBZ); 100% (BPA); 100% (PNP) t_dark_ = 90 min t_light_ = 60 min (TC); 150 min (CBZ); 10 min (BPA); 30 min (PNP)	5 cycles Varies (~100% for PNP to ~83% for CBZ)	[114]

**Table 5:** Summary of MOFs-based MMM’s performance.

MOFs	Matrix	Feeds	PWF (LMH)	Rejection/Removal (%)	FRR (%)	Ref.
**MIL-101** **UiO-66**	PVDF	BSA	360 320	100 98	77.7	[147]
**HKUST-1**	mPES	BSA	490	96	-	[148]
**HKUST-1@GO**	Cellulose acetate	BSA	183.5	91	88.13	[162]
**ZIF-8**	PSU	BSA	298	98.5	81.1	[163]
**MIL-53(Al)**	PSU	Reactive red (RR) Direct Yellow (DY) Methyl Green (MG) Crystal violet (CV) Methylene Blue (MB)	4.8	99.8 99.2 99.5 98.8 97.1	-	[145]
**UiO-66**	Polyethyleneimine (PEI)/PAN	Congo Red (CR)	14.8	99.9		[149]
**ZIF-8**	Cellulose	Rhodamine B (RhB)	14.1	96	>90	[152]
**ZIF-8**	Polyethyleneimine (PI)/hydrolyzed polyacrylonitrile (HPAN)	Methylene Blue (MB) Humic Acid (HA) BSA	33	99.6 -	- 87.8 83.3	[153]
**MOF-2(Cd)**	Polyimide	Methylene Blue (MB) Eosin Y	117.8–171.4	99.9 81.2	-	[164]
**MIL-125(Ti)**	PVDF (photocatalytic membrane)	Rhodamine B (RhB)	64.3	99.7	~100	[150]
**PW12@UiO-66**	PAA-PVA (photocatalytic membrane)	Methyl orange (MO)	-	97.35	-	[157]
**MIL-88B(Fe)**	Al_2_O_3_ hollow (photocatalytic membrane)	Phenol	4000–4500	95	~94%	[160]
**UiO-66@GO**	Palyamide NF (photocatalytic membrane)	Suwannee River humic acid Carbamazepine (CBZ) Diclofenac sodium (DCF)	63	- 70% 93%	97 -	[161]

## Data Availability

Not applicable.
